# The Prevalence of COVID-19 Infection in Students and Staff at a Private University in Thailand by Rapid SARS-CoV-2 Antigen Detection Assay

**DOI:** 10.1155/2022/2350522

**Published:** 2022-03-03

**Authors:** Sunsiree Muangman, Yaowaluk Pimainog, Supachai Kunaratnpruk, Panan Kanchanaphum

**Affiliations:** ^1^Faculty of Medical Technology, Rangsit University, Rangsit, Thailand; ^2^Office of the Vice President for Medical and Health Science, Rangsit University, Rangsit, Thailand; ^3^Biochemistry Unit, Department of Biomedical Science, Faculty of Science, Rangsit University, Rangsit, Thailand

## Abstract

The COVID-19 pandemic led to the suspension of all university courses which was followed directly by the implementation of online learning in Thailand. However, online learning was not suitable for all of Thailand. Rangsit University is a famous private university in Thailand and has been affected by this crisis, so it attempted to eliminate online learning by offering vaccination and antigen rapid screening tests to the students and staff who had to attend the university from July to September 2021. 93.71% of the students and staff from Rangsit University who attended the university from July to September 2021 were vaccinated. Only 1.18% of the students and staff were infected. The vaccines used were CoronaVac and AstraZeneca at 66.02% and 33.98%, respectively. The percentage of individuals that were infected after vaccination did not differ between the two vaccines. The percentage of people infected was 0.31% for CoronaVac and 0.29% for AstraZeneca. Other important factors that influenced the infection rate were the initial symptoms and the environment. Individuals who had initial symptoms and had visited areas with high-risk factors had a high possibility of becoming infected. This research is intended to be useful for risk management during the COVID-19 crisis.

## 1. Introduction

Previously, coronavirus was mostly found in animals and rarely found in humans. Recently, a new strain of coronavirus has been detected in humans. The first patient infected with coronavirus was detected in December 2019 in Wuhan, the capital of China's Hubei province [1]. The coronavirus that caused this disease outbreak belongs to the Coronaviridae family [2], an emerging infectious disease caused by the severe acute respiratory syndrome coronavirus 2 (SARS-CoV-2) [3]. Coronavirus disease 2019 or COVID-19 developed rapidly during 2020 and spread globally to become a pandemic [4, 5].

Thailand was affected by the COVID-19 pandemic. The first COVID-19 patients in Thailand were discovered on 13 January 2020 [6]. Since then, many steps have been taken to contain the outbreak, including a nighttime curfew and the closing of schools and universities. The suspension of regular learning activities at all educational institutions to assist in containing the COVID-19 outbreak was followed directly by the implementation of online learning during the outbreak in Thailand. The ministry of education in Thailand released the following four online learning measures for all universities and educational institutions during the pandemic. The first measure was concerned with preparing for online learning. The second measure was related to testing the online learning system. The third measure involved online learning activities, and the last measure was regarding online examinations. Learning outcomes varied according to the regions of Thailand that were affected by the online learning infrastructure such as Internet accessibility, Internet speed, the online learning platform, and computer accessibility [7].

**Figure 1:**
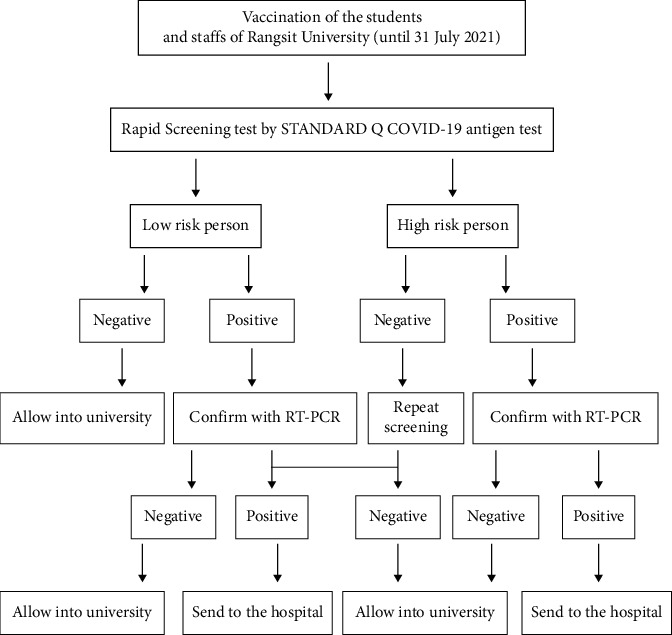
Rangsit University COVID-19 guidelines.

Rangsit University, a leading private university in Thailand, was affected by the pandemic. It implemented COVID-19 measures to reduce the hindrances of online learning for students and staff that enabled them to attend the university campus by offering vaccinations and rapid antigen screening tests, as shown in the Rangsit University COVID-19 guidelines in Figure 1. The vaccines that were given to the students and staff were CoronaVac (Sinovac Life Sciences) and AstraZeneca (AstraZeneca). The STANDARD Q COVID-19 antigen test kit (SD Biosensor, Gyeonggi-do, South Korea) rapid antigen screening test was used.

The real-time reverse transcription-polymerase chain reaction or RT-PCR technique is the current standard test for laboratory diagnosis of SARS-CoV-2 infection in patients. This test requires time and sophisticated equipment and is relatively expensive. Therefore, rapid, easy, inexpensive, and accurate testing for SARS-CoV-2 screening is essential to control disease prevention. Chaimayo et al. [6] demonstrated the effectiveness of the STANDARD Q COVID-19 antigen test kit (SD Biosensor, Gyeonggi-do, South Korea) rapid antigen screening test in preoperative patients at Siriraj Memorial Hospital, Thailand. For this reason, Rangsit University used this test kit for COVID-19 screening.

This is the first report on using the rapid antigen screening test on staff and students to manage the outbreak of COVID-19 at a university in Thailand.

This research explores the prevalence of the COVID-19 infection in students and staff at Rangsit University using results from the STANDARD Q COVID-19 antigen test kit. It is hoped that the results of this study will help decision-makers manage education and other activities at the university during the COVID-19 pandemic.

## 2. Materials and Methods

### 2.1. Study Design

This research is a retrospective study that analyzed the data from questionnaires provided by 2,466 individuals who worked and studied at Rangsit University from 1 July to 30 August 2021 and were screened using the STANDARD Q COVID-19 antigen test kit (SD Biosensor, Gyeonggi-do, South Korea). This test kit has been used and validated by many researchers. Ristic et al. evaluated the performance of this test kit among symptomatic patients during the early and final phases of COVID-19 [8]. Chaimayo et al. [6] compared the efficiency of this test kit with real-time RT-PCR testing in patients at Siriraj Memorial Hospital, Thailand. They found that the sensitivity of this test kit was 98.33% (98% CI, 91.06–99.0). The information collected was gender, the status of the person, vaccination, type of vaccine, initial symptoms, and the environment. The Faculty of Medical Technology at Rangsit University was responsible for the screening test and collecting the questionnaires. This study was approved by the Ethics Review Board of Rangsit University (DPE.No. RSUERB2021-019).

### 2.2. Statistical Analysis

Descriptive statistical analyses were performed using frequency and percentage. The prevalence of the infection was analyzed by the chi-square test. All analyses were performed using SPSS version 25, and a *p* value of less than or equal to 0.05 was considered statistically significant.

## 3. Results

**Figure 2:**
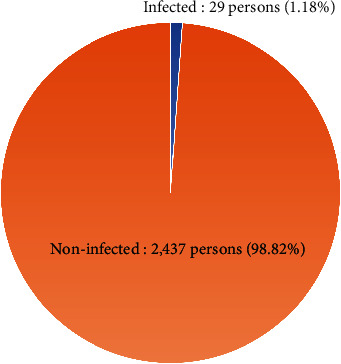
Results of rapid screening tests from July to August 2021.

From the rapid screening tests administered from July to August 2021, 2,437 individuals tested negative and only 29 (1.18%) individuals were infected, as shown in Figure 2. All the infected individuals had their results confirmed by the real-time reverse transcription PCR (real-time RT-PCR) test.

The characteristics of the 2,466 individuals are shown in Table 1.

Characteristics such as the status of individuals that passed the screening test and the number of infected individuals are shown in Table 2.

An important characteristic of the individuals who passed this screening test was their vaccination status, as shown in Table 3. The results show that the vaccination status was statistically significant with an infection rate at the 0.05 level.

Another characteristic considered was the type of vaccine. The vaccines that were given to the students, lecturers, and officers from Rangsit University were CoronaVac and AstraZeneca. The relationship between the type of vaccine and the infection rate is shown in Table 4. It shows that there was no difference in the infection rate between individuals that received the CoronaVac or AstraZeneca vaccine.

Notable interesting characteristics are the initial symptoms and the environment. The relationships between the initial symptoms, the environment, and the infection rate are shown in Table 5. The results show that the initial symptoms and environment were statistically significant with an infection rate at the 0.05 level.

## 4. Discussion

**Figure 3:**
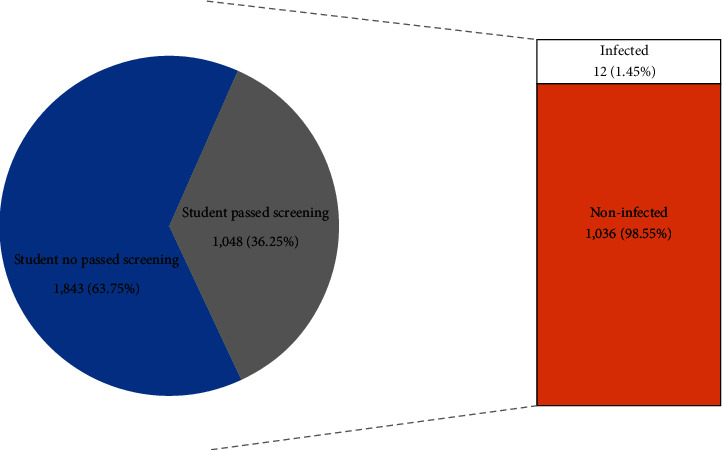
The percentages of the students who passed and did not pass the rapid screening test.

The majority who passed this screening test were students and officers at 42.50% and 44.85%, respectively, as shown in Table 3. The students who passed the screening test were mostly registered in the summer semester of 2021 (June–August 2021). The number of registered students in the summer semester of 2021 was 2,891 [9]. The percentage of students who passed the screening test was 36.25% or more than a third of the total students who registered in the summer semester of 2021, as shown in Figure 3. Only 12 students were found to be infected equal to 1.45% of the students who took the screening test, as shown in Figure 3. These results indicate that the students at Rangsit University were careful and had practiced self-protective measures such as wearing masks and social distancing. Consequently, the infection rate was quite low. Conversely, Blake et al. [10] reported that 48% of students were tested for COVID-19 infection. The percent of the total students in that study was higher than that in this study because Blake et al. analyzed only 25 students. As this was a smaller sample, it may not be as accurate. In our study, there were 1,048 students or more than a third of the students who registered for the summer semester of 2021. No lecturer who took this screening test was infected. This implies that the lecturers may understand how to effectively prevent COVID-19 infection. Table 3 shows that 17 officers were infected. It is interesting to note that all the infected individuals had one or both of the following characteristics: symptoms of hypertension or diabetes (data not shown) and middle age. These results concur with Liu et al. [11] who concluded that the major diseases which are more susceptible for COVID-19 in middle-aged and elderly people are diabetes, hypertension, cardiovascular disease, and cerebrovascular disease.

There were 2,311 individuals or 93.71% who were vaccinated, and only 7 individuals or 0.3% were infected. When compared with the individuals who were not vaccinated, 155 individuals or 6.29%, there were 22 infected individuals (14.47%) in this group. 93.71% of those who passed this screening test were vaccinated. This suggests that most students and staff attending Rangsit University understood and had become aware of the importance of vaccination. These results imply that vaccinations can protect against infection with a statistical significance similar to the research results from many other countries such as Turkey, Chile, Indonesia, and Brazil [12]. This was more than 50% (84% in Turkey, 67% in Chili, 65% in Indonesia, and 51% in Brazil) [12].

Almost all individuals (2,016 people or 81.75%) who passed the screening test were not at-risk individuals, and 8 people or 0.4% were infected. 450 individuals who passed the screening test were at-risk individuals and 21 or 4.66% of them were infected, which shows that the environment is statistically significant to the infection rate. These results concur with Doung-ngern et al. [13] who showed that at-risk individuals have a higher probability of becoming infected. However, the number of infected individuals from at-risk environments in our study is lower than that was found by Doung-ngern et al. [13]. This may be because all the individuals who passed the screening test were students, lecturers, and staff from the university. Their chances of visiting at-risk areas during the pandemic were lower than those of the people who work and live in these areas. There were an interesting number of COVID-19 cases associated with at-risk areas, especially nightclubs in Bangkok. About 16.6% of infected individuals had visited nightclubs [13] according to the number of COVID-19 cases found at the Itaewon nightclub cluster in Seoul, South Korea, in May 2020 [14]. These individuals visited several nightclubs in the same area during a short period. The infection rate at a boxing stadium in Bangkok was high (86%) [12], which was similar to the cluster of COVID-19 cases associated with a football match in Italy in February 2023 [15]. Therefore, it can be concluded that the environment plays a statistically significant role in the infection rate. Deiana et al. [16] reported that the infection rate among healthcare professionals within residential care homes and healthcare facilities who had a high risk of contacting COVID-19 patients was a significant concern.

Initial symptoms were another influential factor. From the 208 individuals that passed the screening test who displayed initial symptoms (cough, sore throat, tasteless tongue, anosmia, and dyspnea), 15 individuals (7.21%) were infected and 14 of those infected individuals had not been vaccinated. This confirms that vaccinations can protect against virus infection. The infection rate (7.21%) found in this research concurs with Torres et al. [17] who studied 634 individuals that were in close contact with infected patients. They used the rapid antigen test (Panbio^TM^ COVID-19 Ag rapid test device) and confirmed their results using real-time testing (RT-PCR). They found that there were 38 infected persons or 5.99% who had initial symptoms.

An important factor was the type of vaccine. In this study, the individuals who passed the screening test were given either the CoronaVac or AstraZeneca vaccination.

The CoronaVac vaccine administered was the CoronaVac inactivated SARS-CoV-2 vaccine. This is a chemically inactivated, whole SAR-CoV-2 preparation [18]. This vaccine is being evaluated in Phase I/II/III trials in Brazil and China in both adults and geriatric parenteral, i.e., intramuscular (*i.m.*) [19,20]. No serious local and systemic reactions to the vaccine were observed [19]. It was observed that the neutralizing antibody titers were comparatively higher in younger patients when compared to older ones and the second dose kinetics yielded different responses, i.e., stronger immune responses with the second dose on the 28^th^ day instead of the 14^th^ day [19,21].

The AstraZeneca vaccine was developed by the University of Oxford and the Serum Institute of India. It is based on the nonreplicating “ChAdOx1” vector that was previously termed as “ChAdOx1 nCoV-19” and is now known as “AZD1222” [22,23]. The AZD1222 vaccine expresses a full-length unmodified wild-type version of the S (spike) protein [23]. The advantage of the ChAdOx1 vector-based vaccine over commonly used human Ad5 (hAd5) vector-based vaccines is that it is primate-derived, originating from chimpanzees. The route of administration is parenteral, i.e., intramuscular (*i.m.*), and it is being evaluated as a single- or two-dose regimen in Phase III clinical trials in several countries. The vaccine had mild adverse reactions including chills, fatigue, headache, fever, nausea, muscle aches, malaise, and painful injection sites within a week of vaccination [22–26].

This study can help to understand the prevalence and epidemiology of COVID-19 on the campus of a university. This research may be used as a model for other universities to initiate guidelines or policies to prevent COVID-19 from spreading. This is in agreement with Deiana et al. [16] who concluded that understanding the epidemiology and transmission dynamics of the epidemic outside of semiclosed communities would provide appropriate information to guide intervention policy. The university campus is an open area that shares the same air, water, and facilities which may result in the transmission of the virus among staff and students. Asymptomatic people can come and go without limitations, causing the virus to spread around the university campus and into the community [27]. Therefore, COVID-19 screening for individuals that attend the university is a necessary measure to prevent the virus from spreading.

**Figure 4:**
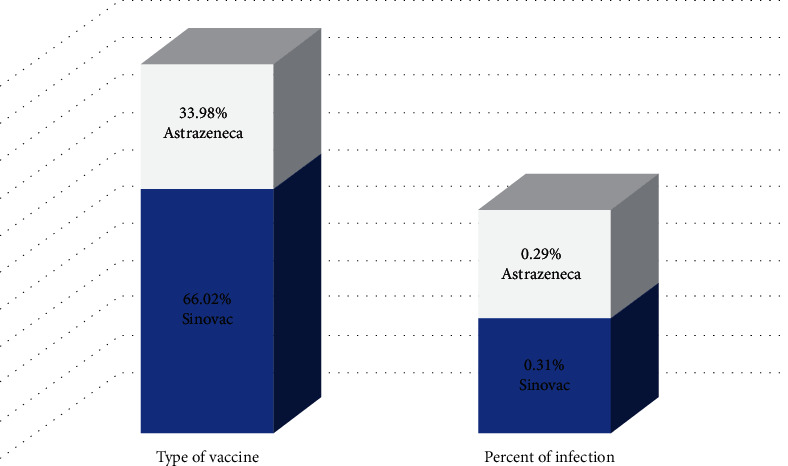
The percent of vaccinated and infected individuals after vaccination.

In our study, most individuals who passed the screening test were given CoronaVac (66.02%) and 33.98% were given AstraZeneca. However, the percentage of individuals who were infected after vaccination was not different, as shown in Figure 4. Figure 4 indicates that individuals who were inoculated with either CoronaVac or AstraZeneca had an equal chance of infection. However, individuals who have already been vaccinated must be careful and still take protective measures to reduce the risk of infection.

This is the first study that explores the prevalence of COVID-19 infection in staff and students attending a university in Thailand. Universities must create guidelines or policies to manage and prevent potential outbreaks of COVID-19 on the campus and manage education and other activities at the university during the COVID-19 pandemic.

This study has several limitations. Firstly, individuals who received one dose or two doses of the vaccine were not identified, which could affect the efficiency of the vaccine. Second, the students' field of study is not categorized. Some students such as those from the Faculty of Nursing Science and the Faculty of Physical Therapy are required to study on site in the laboratory, and they come into contact with many people. This group has a higher risk of catching and spreading the virus. However, other students such as those from the Faculty of Accounting can study online. Considering these data may help to manage the schedules of students who are at a higher risk of spreading and catching the virus. Finally, the reasons why some individuals did not get vaccinated were not investigated.

## 5. Conclusion

Rangsit University implemented measures to reduce the impact of online learning. This permitted the students and staff to resume their activities on the university campus. The students and staff attending the university were vaccinated. Before entering the university, a rapid screening test was given to the students and staff. The results of this research showed that almost all individuals who passed the screening test were vaccinated and not infected. However, these guidelines did not completely ensure that COVID-19 did not spread. The measures were useful for managing the risks related to COVID-19 and allowing activities to continue during the COVID-19 crisis.

## Figures and Tables

**Table 1:** Characteristics of the individuals that took the rapid screening test from July to August 2021.

Characteristics	Number of individuals that were rapid screened	Percent
*Gender*		
Male	778	31.55
Female	1688	68.45
*Status*		
Students	1,048	42.50
Lecturers	312	12.65
Officers	1,106	44.85
*Vaccination*		
Vaccinated	2,311	93.71
Nonvaccinated	155	6.29
*Type of vaccine*		
CoronaVac	1,628	66.02
AstraZeneca	683	33.98
*Initial symptoms*		
Symptomatic	208	8.43
Asymptomatic	2,258	91.57
*Environment*		
Risky	450	18.25
Nonrisky	2,016	81.75

**Table 2:** Status of the individuals who passed the screening test and the number of infected individuals.

Status	Number	Percent	Infected (%)
Students	1,048	42.50	12 (1.45%)
Lecturers	312	12.65	0 (0%)
Officers	1,106	44.85	17 (1.54%)

**Table 3:** Vaccination status of the individuals that passed the screening test and the number of infected individuals.

Status	Number	Percent	Infected (%)
Vaccinated	2,311	93.71	7 (0.30%)^*∗*^
Nonvaccinated	155	6.29	22 (14.47%)^*∗*^

^∗^ *p* value less than or equal to 0.05.

**Table 4:** The relationship between the type of vaccine and the infection rate.

Type of vaccine	Number	Percent	Infected (%)
CoronaVac	1,628	66.02	5 (0.31%)
AstraZeneca	683	33.98	2 (0.29%)

**Table 5:** The relationship between the initial symptoms, the environment, and the infection rate.

Initial symptom		Infected (%)	Noninfected
Symptomatic	208 cases (8.43%)	15 (7.21%)^*∗*^	193
Nonsymptomatic	2,258 cases (91.57%)	14 (0.62%)	2,244
Environment			
Risky	450 cases (18.25%)	21 (4.66%)^*∗*^	429
Not at risk	2,016 cases (81.75%)	8 (0.4%)	2,008

^∗^ *p* value less than or equal to 0.05.

## Data Availability

The data used to support the findings of this study are available from the corresponding author upon request.
